# Mesenchymal stem cells protect podocytes from apoptosis induced by high glucose via secretion of epithelial growth factor

**DOI:** 10.1186/scrt314

**Published:** 2013-09-01

**Authors:** Diangeng Li, Nan Wang, Li Zhang, Zhu Hanyu, Bai Xueyuan, Bo Fu, Cui Shaoyuan, Weiguang Zhang, Sun Xuefeng, Rongshan Li, Xiangmei Chen

**Affiliations:** 1 Department of Nephrology, State Key Laboratory of Kidney Diseases, Chinese PLA General Hospital and Medical School of Chinese PLA, 28# Fu Xing Road, Beijing 100853, China; 2 Department of Nephrology, The Second Hospital of Shanxi Medical University, 382# Wu Yi Road, Tai Yuan, Shanxi 030001, China

**Keywords:** Apoptosis, Diabetic nephropathy, Epithelial growth factor, Injury, Mesenchymal stem cells, Podocyte

## Abstract

**Introduction:**

The apoptosis and subsequent injury of podocytes plays a pathogenic role in diabetic nephropathy (DN). Mesenchymal stem cells (MSCs) are promising therapeutic cells for preventing apoptosis and reducing cellular injury. Our previous study found that MSCs could protect kidneys from diabetes-induced injury without obvious engraftment. So we evaluated the effects of human adipose-derived MSCs (hAd-MSCs) on podocytic apoptosis and injury induced by high glucose (HG) and the underlying mechanisms.

**Methods:**

We used flow cytometry, Western blot and confocal fluorescence microscopy to study podocytic apoptosis and injury induced by HG at 24 hours, 48 hours, and 72 hours in the presence or absence of MSC-conditioned medium (CM). An antibody-based cytokine array was used to identify the mediating factor, which was verified by adding the neutralizing antibody (NtAb) to block its function or adding the recombinant cytokine to the medium to induce its function.

**Results:**

hAd-MSC-CM reduced podocytic apoptosis in a dose-dependent manner, decreased the expression of podocytic cleaved caspase-3, and prevented the reduced expression and maintained the normal arrangement of podocytic synaptopodin and nephrin. However, human embryonic lung cell (Wi38)-CM failed to ameliorate podocytic apoptosis or injury. Twelve cytokines with concentration ratios (MSC-CM/Wi38-CM) >10-fold were identified. Epithelial growth factor (EGF) was singled out for its known ability to prevent apoptosis. Recombinant human EGF (rhEGF) prevented podocytic apoptosis and injury similarly to hAd-MSC-CM but, upon blockade of EGF, the beneficial effect of hAd-MSC-CM decreased dramatically.

**Conclusions:**

hAd-MSCs prevent podocytic apoptosis and injury induced by HG, mainly through secreting soluble EG.

## Introduction

Diabetic nephropathy (DN) is one of the most common and serious complications of capillaries in patients with diabetes [1]. In recent years, research on the structure and function of the filtration barrier of the glomerulus has focused on the function of podocytes in the occurrence and development of DN [2]. Podocytes are terminally differentiated epithelial cells attached to the glomerular basement membrane, and the alternative podocytic processes between adjacent podocytes construct the slit diaphragm, which plays an important role in maintaining the integrity of the filtration barrier structure and function of the glomerulus [3-5]. High glucose contributes to reduced podocyte number and induces apoptosis of cultured podoctes and causes albuminuria and accelerates foot process effacement [2,3,6,7]. At present, there is no satisfactory method or target for the treatment of DN.

Mesenchymal stem cells (MSCs), a group of cells from the mesoblast that have characteristics of stem cells, show strong proliferation ability and multipotential differentiation, which can repair injured tissues and cells by secreting cytokines [8-10]. Treatments involving MSCs may be effective for repairing injured tissues and cells clinically [8,11]. It has been shown that MSCs can promote tissue repair through chemotaxis and homing to the damaged location *in vivo*, or through the paracrine action of various cytokines, to reduce adverse reactions of the kidney, so as to improve and promote the endogenous repair of kidney tissue [12-15]. It is easy to obtain MSCs from adipose tissue, which has stable cellularity. After high passage of these cells, only a low level of senescence will occur [16]. These cells have the ability to secrete a large number of protective cytokines [17,18] and show other MSCs characteristics, such as self-renewal and multiple lineage differentiation. In preliminary experiments, we found that human adipose-derived (hAd)-MSCs injected via the tail vein into rats with DN alleviated kidney injury, reduced proteinurea, and prevented the downregulation of synaptopodin (data not shown); however, the gross presence of stem cells was not found in the kidney. Thus, we speculated that hAd-MSCs protected the kidney via paracrine action.

Using hAd-MSC-conditioned medium (CM) with a model of podocytic apoptosis induced by high glucose (HG), we investigated whether MSCs-CM could itself inhibit HG-induced podocytic apoptosis, to identify the effect or molecule(s) and to provide novel insights into the treatment of DN.

## Methods

### Cell culture and processing

A mouse podocyte clone 5 (MPC5), which was provided by Professor Peter Mundel of the Medical College of Harvard University (Boston, MA, USA), was cultured as previously described [16,19]. Briefly, cryopreserved podocytes were cultured and amplified in 11 mM D-glucose Roswell Park Memorial Institute (RPMI)-1640 medium containing 20 units/ml IFN-γ (Invitrogen, Carlsabad, CA, USA) and 10% FCS at 33°C. After passage at 37°C, the podocytes were cultured in IFN-γ-free medium for 10 days (changing the solution every two days) to induce differentiation. The presence of synaptopodin and nephrin indicated podocytic differentiation and was detected by confocal microscopy. The cells were cultured synchronously in medium containing 0.2% FCS and 5.5 mM D-glucose RPMI-1640 medium for 24 hours before the experiment. MPC5 cells were divided mainly into seven groups according to treatment: normal glucose (NG, 5.5 mM), mannitol control (NG+Ma, 5.5 mM D-glucose + 24.5 mM mannitol), HG (30 mM), treatment group in human embryonic lung cells (Wi38)-CM (1×, 2×, 4×), treatment group in MSC-CM (1×, 2×, 4×), recombinant human epidermal growth factor (rhEGF, 3 ng/ml) treatment group, and MSC-CM neutralizing antibody (MSC-CM+EGF+NtAb) group.

hAd-MSCs were provided by the Microcirculation Institute of the Chinese Academy of Medical Sciences (Beijing, China). These cells from patients undergoing selective suction-assisted lipectomy were collected after obtaining informed consent from the patients according to procedures approved by the Ethics Committtee of the Chinese Academy of Medical Sciences and Peking Union Medical College. The adipose tissue was extensively washed with D-Hanks’ solution to remove contaminating blood cells and local anesthetics. Then, it was resuspended in 0.075% typeΙA collagenase (Sigma-Aldrich Corp, St. Louis, MO, USA)/Hank's Balanced Salt Solution (approximately 2 ml/g) and incubated at 37°C for one hour to release the cellular fractions. The digested adipose tissue was passed through a 100-μm filter to remove debris and centrifuged at 150 × g for 10 minutes to produce a cell pellet. After isolation, 30 ml of resuspended cells were plated in expansion medium at a density of 5 to approximately 6 × 10^6^ nucleated cells/75 cm^2^ tissue culture dish and incubated at 37°C. Once adherent cells were more than 80% confluent, they were detached with 0.125% trypsin and 0.01% ethylenediaminetetraacetic acid (EDTA) and replated at a 1:4 dilution under the same conditions according to previous protocols [16,20]. All the experiments were done with the 5th passage and the 20th passage and the 5th passage of hAd-MSCs was used for the results given in this article.

Wi38 were purchased from the American Type Culture Collection (ATCC, Manassas, VA, USA) and were cultured in RPMI-1640 medium containing 10% FCS and passaged when confluence reached 80%.

### Preparation of CM

hAd-MSCs and Wi38 cells were cultured in their respective media at a concentration of 1 × 10^5^ cells/cm^2^ and were rinsed three times in PBS after one day of cell adhesion. Then, the cells were cultured in serum-free RPMI-1640 medium for 48 hours and the culture supernatants were collected. The supernatants were centrifuged at 1,500 rpm for 10 minutes at 4°C and transferred to a centrifugal column with a 3 kDa cut-off (Millipore, Billerica, MA, USA). This resulted in 15× MSC-CM or Wi38-CM, respectively, which were desalted according to the manufacturer’s protocol. CM was then sterilized by filtration through a 220 nm filter (Millipore, Billerica, MA, USA).

### The expression of cleaved caspase-3 in MPC5 cells detected by Western blot

MPC5 cells were collected, and the total protein was extracted. The protein concentration was determined by BCA assay (Pierce, Rockford, IL, USA), and 2× SDS-denatured protein (95°C for 5 minutes and ice-bath for 10 minutes) in the same volume was added. The total protein of podocytes was isolated using 15% SDS-PAGE and 80 μg of each sample. The isolated protein was transferred to a nitrocellulose membrane. The primary rabbit polyclonal anti-cleaved caspase-3 antibody (Cell Signaling Technology, Beverly, MA, USA) was diluted 1:1000 and incubated with the membrane(s) overnight at 4°C. Then the secondary anti-rabbit antibody (Santa Cruz Biotechnology, Santa Cruz, CA, USA), conjugated to horseradish peroxidase (HRP) and diluted to 1:1000, was added for one hour at room temperature. β-actin was used as the loading control and the blots were developed with an ECL Western Blotting kit (Applygen Technologies Inc, Beijing, China).

### Podocytic apoptosis detected by flow cytometry

After the podocytes treated by different methods were cultured at 37°C for 24, 48, and 72 hours, respectively, and were digested with 0.25% EDTA-free pancreatin (Gibco, Grand Island, NY, USA), they were rinsed twice in PBS. The cell concentration was adjusted to 1 × 10^6^ cells/L to make a single cell suspension. An annexin V/propidium iodide (PI) double staining apoptosis detection kit (BD Biosciences, Franklin Lakes, NJ, USA) was used to stain the podocytes according to the manufacturer’s instructions. The data were acquired on a flow cytometer (Beckman Coulter Inc, CA, USA).

After the podocytes treated by different methods were cultured at 37°C for 24, 48, and 72 hours, respectively, the cell concentration was adjusted to 1 × 106 cells/L to make a single cell suspension. When the apoptosis models were established, reflecting the degree of apoptosis, was measured using flow cytometry with TUNEL staining. An In Situ Cell Death Detection Kit (Roche, Mannheim, Germany) was used to stain podocytes according to the manufacturer’s instructions. The data were also acquired on a flow cytometer (Beckman Coulter Inc).

### Cellular immunofluorescence

Differentiated and matured podocytes were seeded onto six-pore plates and covered by a cover glass that was pretreated with collagen I (Sigma-Aldrich, St. Louis, MO, USA). Each group was subjected to its own designated treatment regimen, and the cells were cultured at 37°C for 24, 48 and 72 hours, respectively. Three accessory foramina were set up for each group at each time point. The cells were fixed with 4% paraformaldehyde for 5 minutes at room temperature followed by incubation at 4°C for 10 minutes. Then, 0.2% Triton X-100 was added to permeabilize the cells for 5 minutes at room temperature and 1× casin was sealed up for 30 minutes at room temperature. Primary rabbit polyclonal antibodies specific for synaptopodin and nephrin were diluted 1:50 and incubated with the cells (Santa Cruz Biotechnology) (ProSci Incorporated, San Diego, California, USA) overnight at 4°C. A secondary anti-rabbit antibody conjugated to Cy3 (Jackson ImmunoResearch Laboratories, Bar Harbor, ME, USA) (diluted 1:1000) was incubated with the cells away from light for one hour at room temperature. Finally, a fluorescent sealing liquid (ZSGB-BIO, Beijing, China) containing 4',6-diamidino-2-phenylindole (DAPI) was added, and confocal laser scanning microscopy (FluoView FV1000; Olympus America Inc., Center Valley, PA, USA) was used to determine the expression level and structure arrangement of podocytic synaptopodin and nephrin. Ten visual fields were observed at random in each culture pore.

### Antibody chip

A cytokine detection kit (RayBio Human Cytokine Array; RayBiotech, Inc., Norcross, GA, USA) was used to determine the presence of cytokines in the 15× concentrated blank RPMI-1640 medium, Wi38-CM, and Ad-MSCs-CM. The analysis was performed using the manufacturer’s recommended protocol, and the signals of Cy3 were imaged by Axon GenePix laser scanner (MDS Analytical Technologies, Sunnyvale, CA, USA). The result was normalized to the respective positive control. Results were obtained from three independent samples.

### Cytokines detected by enzyme-linked immunosorbent assay (ELISA)

The concentration of EGF, insulin-like growth factor binding protein (IGFBP)-1, glial cell line-derived neurotrophic factor (GDNF) and placental growth factor (PIGF) was determined in each culture supernatant (RPMI-1640 medium, Wi38-CM and Ad-MSC-CM) by ELISA kits (R&D Systems, Minneapolis, MN, USA) according to the manufacturer’s instructions. Optical density (OD) was measured at 450 nm and 570 nm. Concentration analysis software (Microplate Manager 4.0) was used to calculate the concentration of candidate factors in the samples according to standard curves. Three independent samples were placed in three repetitive holes.

### Recombinant factor and NtAb experiments

To prepare rhEGF, rhIGFBP-1, rhGDNF and rhPIGF, the respective lyophilized powders (PeproTech, RockyHill, NJ, USA) were reconstituted to a working concentration (0.2 mg/ml) with sterile, deionized water and cryopreserved at −20°C.

A solution of rhEGF NtAb (PeproTech, Rocky Hill, NJ, USA) was diluted using the respective CM to a ratio (EGF: EGF-NtAb) of 1 ng/ml : 0.175 μg/ml and was incubated at 37°C for one hour before using.

### Statistical analysis

Analysis was done using SPSS 17.0 software. Results are presented as mean ± standard deviation. One-factor analysis of variance (one-way ANOVA) was used to compare differences among groups. *P* <0.05 was considered statistically significant for all analyses.

## Results

### Podocytic apoptosis and injury was induced by HG

**Figure 1:**
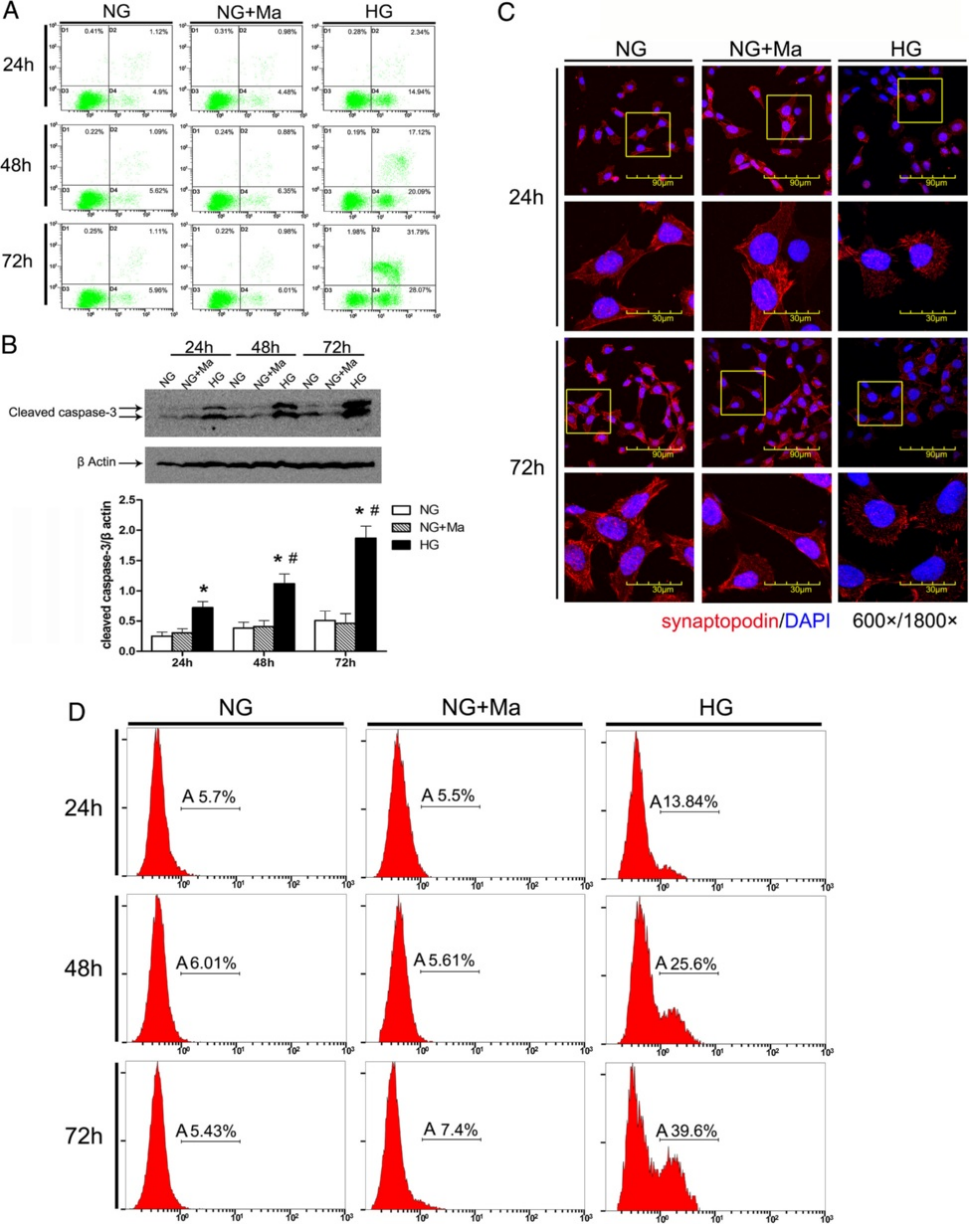
**High glucose (HG) induces apoptosis and injury of mouse podocyte clone (MPC5) cells. A)** AnnexinV/PI double-staining-labeled cells in each group (n = 3 per group). The number of apoptotic or necrotic cells was quantified by FACS analysis after staining with annexin V and PI. The cytograms show viable cells that did not bilnd annexin V or PI in the D3 quadrant. Cells at early stages of apoptosis that bound annexin V but that still had intact cell membranes and excluded PI are shown in the D4 quadrant. Cells with advanced stages of apoptosis or necrotic were both annexin V and PI positive and are shown in the D2 quadrant. Cells lost its intact cell membranes that bound PI and excluded annexin V are shown in the D1 quadrant. The results showed that podocytic apoptosis rate was significantly higher at all time points in HG group than in normal glucose (NG) group, and was time-dependent. **B)** Western blot was used to detect the expression of cleaved caspase-3 at three time points (24, 48 and 72 hours). The expression of cleaved caspase-3 was increased with the prolonged stimulation of HG. All of the experiments were repeated three times (n = 3). *P <0.05, HG group *versus* NG group or NG+mannitol group; ^#^*P* <0.05, 48-hour HG group or 72-hour HG group *versus* 24-hour HG group. **C)** The expression and the location of podocytic cytoskeletal protein synaptopodin (red) were measured by confocal microscopy. The expression of podocytic synaptopodin in the HG group was reduced and rearranged. Nuclei were stained with DAPI (blue). Magnification = 600×, 1800×. **D)** Using flow cytometry with TUNEL staining to measure the apoptosis rate of podocytes under treatment with NG, NG+Ma and HG at three time points (24, 48 and 72 hours) (n = 3 each group). Cells analyzed under marker ‘A’ are apoptotic (TUNEL positive).

MPC5 cells were cultured *in vitro* and glucose (30 mM) was added to induce apoptosis to establish a model of podocytic apoptosis and injury. AnnexinV/PI double staining and flow cytometry were used to detect podocytic apoptosis, and the results showed that podocytic apoptosis rates were significantly higher at all time points in the HG group than in the NG group and were time-dependent (*P* <0.05) (Figure 1A). Western blot was used to detect cleaved caspase-3. The expression of cleaved caspase-3 increased more with the prolonged stimulation HG (P <0.05) (Figure 1B). Confocal immunofluorescence was used to detect the expression of synaptopodin (one of podocytic skelemins), and the results showed that the expression of podocytic synaptopodin in the HG group was reduced and rearranged, while these changes did not occur in the NG+Ma group (Figure 1C). The data suggest that podocytic apoptosis and injury was induced by the increased concentration of glucose, which was aggravated with prolonged stimulation time.

### hAd-MSC-CM reduced podocytic apoptosis and injury induced by HG

**Figure 2:**
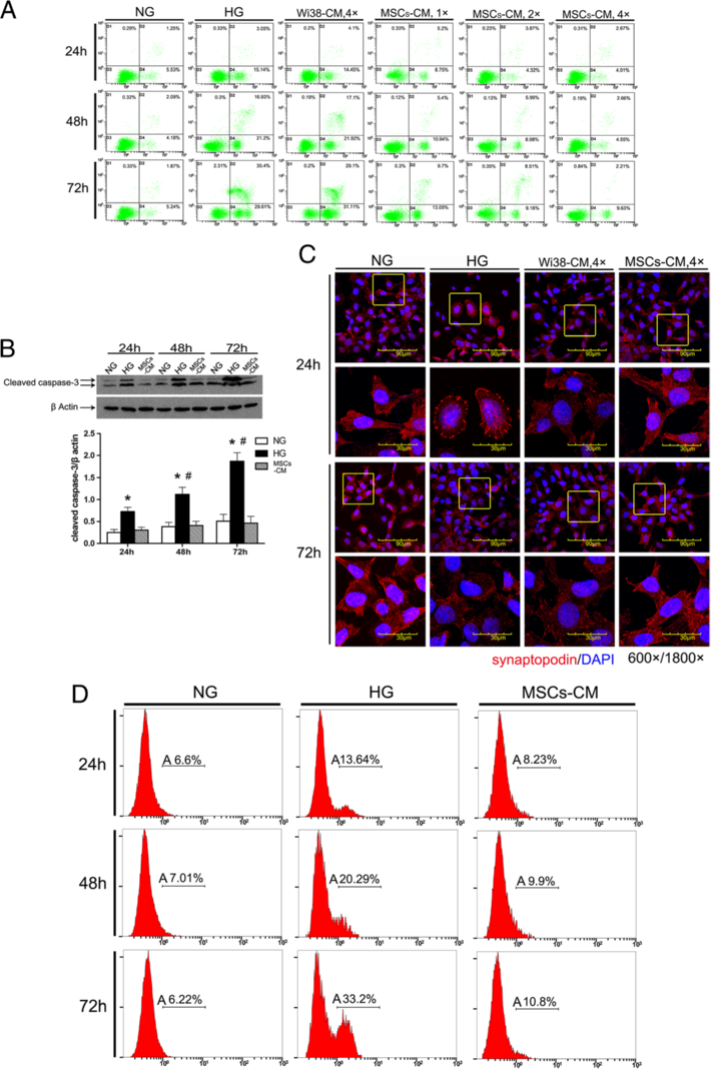
**Human adipose-derived mesenchymal stem cells (hAd-MSCs)-conditioned medium (CM) reduces apoptosis and injury of mouse podocyte clone (MPC5) cells induced by high glucose (HG). A)** Representative photographs of annexinV/PI double-staining in different groups (n = 3 per group) and flow cytometry to test the podocytic apoptosis rate after culture in Wi38-CM or MSC-CM. MSC-CM reduced podocytic apoptosis in a dose-dependent manner, but Wi38-CM did not. **B)** Western blot was used to detect the expression of cleaved caspase-3 at three time points (24, 48 and 72 hours). MSC-CM reduced the expression of cleaved caspase-3, but Wi38-CM did not. All of the experiments were repeated three times (n = 3). *P <0.05 HG group *versus* normal glucose (NG) group or MSC-CM group; #*P* <0.05, 48-hour HG group or 72-hour HG group *versus* 24-hour HG group. **C)** The expression and location of the podocytic cytoskeletal protein synaptopodin (red) were measured by confocal microscopy. MSC-CM prevented the downregulation and rearrangement of synaptopodin, but Wi38-CM did not. Nuclei were stained with DAPI (blue). Magnification = 600×, 1800×. **D)** Using flow cytometry with TUNEL staining to measure the apoptosis rate of podocytes under treatment with NG, HG and MSCs-CM at three time points (24, 48 and 72 hours) (n = 3 each group). Cells analyzed under marker ‘A’ are apoptotic (TUNEL positive). The results showed that the podocytic apoptosis rate was significantly lower at all time points in the MSCs-CM group than in the HG group. DAPI, 4',6-diamidino-2-phenylindole; PI, propidium iodide.

After establishing a model of podocytic apoptosis and injury induced by HG *in vitro*, we used MSC-CM to culture podocytes in HG. hAd-MSCs can be easily harvested from patients in a simple, minimally invasive lipoaspiration procedure and can be expanded *in vitro*[20]. Both hAd-MSCs and Wi38 are human fibroblasts, but Wi38-CM does not have the characteristics of hAd-MSCs. As a kind of stem cell, hAd-MSCs have the characteristics of self-renewal and multi-differentiation. In addition, hAd-MSCs have a characterized cytokine secretion profile which Wi38 does not. So, we used Wi38-CM as the control [21,22]. Compared with the HG group, hAd-MSC-CM reduced podocytic apoptosis induced by HG in a dose-dependent manner (*P* <0.05) (Figure 2A and D), downregulated activated caspase-3 (*P* <0.05) (Figure 2B), and prevented the downregulation and rearrangement of synaptopodin (Figure 2C). However, in the Wi38-CM treatment group, there was no significant improvement in these same measures (Figure 2B). Consequently, MSC-CM could prevent podocytic apoptosis induced by HG and reduce the injury of podocytic skelemins, but Wi38-CM could not. Therefore, it was the cytokines present in MSC-CM but not in Wi38-CM that contributed to protecting podocytes from the influence of HG.

### Screening of candidate factors in MSC-CM

We used an antibody-based cytokine array to detect the cytokines in MSC-CM and Wi38-CM and selected the following cytokines with MSC-CM/Wi38-CM >10: basic fibroblast growth factor (bFGF) (3,381-fold), EGF (117-fold), IGFBP-1 (45-fold), GDNF (30-fold), and PIGF (123-fold).

It was reported that bFGF could augment podocyte damage, resulting in increased glomerular protein permeability and accelerated glomerulosclerosis [23]. We used ELISA kits to measure the concentration of cytokines in CM: EGF (MSC-CM, 3.81 ± 0.18 ng/ml; Wi38-CM, 29 ± 1.1 pg/ml), IGFBP-1 (MSC-CM, 0.98 ± 0.01 ng/ml; Wi38-CM, 21.7 ± 1.9 pg/ml), GDNF (MSC-CM, 3.37 ± 0.12 ng/ml; Wi38-CM, 119 ± 21 pg/ml), and PIGF (MSC-CM, 1.1 ± 0.09 ng/ml; Wi38-CM, 45.8 ± 2.2 pg/ml).

**Figure 3:**
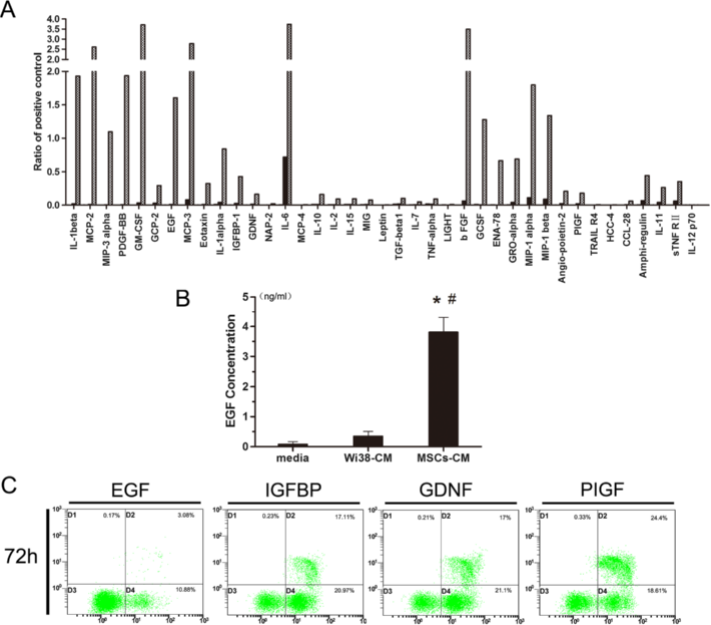
**Screening candidate effector factors in human adipose-derived mesenchymal stem cells (hAd-MSCs)-conditioned medium (CM). A)** Cytokine microarray to determine the soluble cytokines present in blank medium, Wi38-CM and MSC-CM. Cytokines of MSC-CM/Wi38-CM >10: basic fibroblast growth factor (bFGF) (3,381-fold), epithelial growth factor (EGF) (117-fold), insulin-like growth factor binding protein (IGFBP)-1 (45-fold) and glial cell line-derived neurotrophic factor (GDNF) (30-fold). The data were normalized to the respective positive controls. **B)** The concentration of EGF in CM was determined by ELISA. MSC-CM: 3.81 ± 0.18 ng/ml; Wi38-CM: 29 ± 1.1 pg/ml. *P <0.05 Wi38-CM or MSC-CM *versus* blank medium; #*P* <0.05, MSC-CM *versus* Wi38-CM. **C)** AnnexinV/PI double staining labeled cells in each group (n = 3 per group) and flow cytometry to determine the podocytic apoptosis rate. Growth factor (EGF) significantly reduced podocytic apoptosis,but other factors (IGFBP, GDNF, PIGF) did not. PI, propidium iodide.

Based on these results, we prepared one-component culture medium to podocytes in HG and used flow cytometry to detect podocytic apoptosis. Results showed that rhEGF (3.5 ng/ml) effectively inhibited podocytic apoptosis induced by HG (*P* <0.05) compared with the HG group, but rhIGFBP, rhGDNF, or rhPIGF (1 ng/ml, 3 ng/ml, 1 ng/ml) could not (Figure 3C). Therefore, we chose EGF as the candidate cytokine for further investigation (Figure 3).

### hAd-MSCs reduce podocytic apoptosis and injury induced by HG through secreting EGF

**Figure 4:**
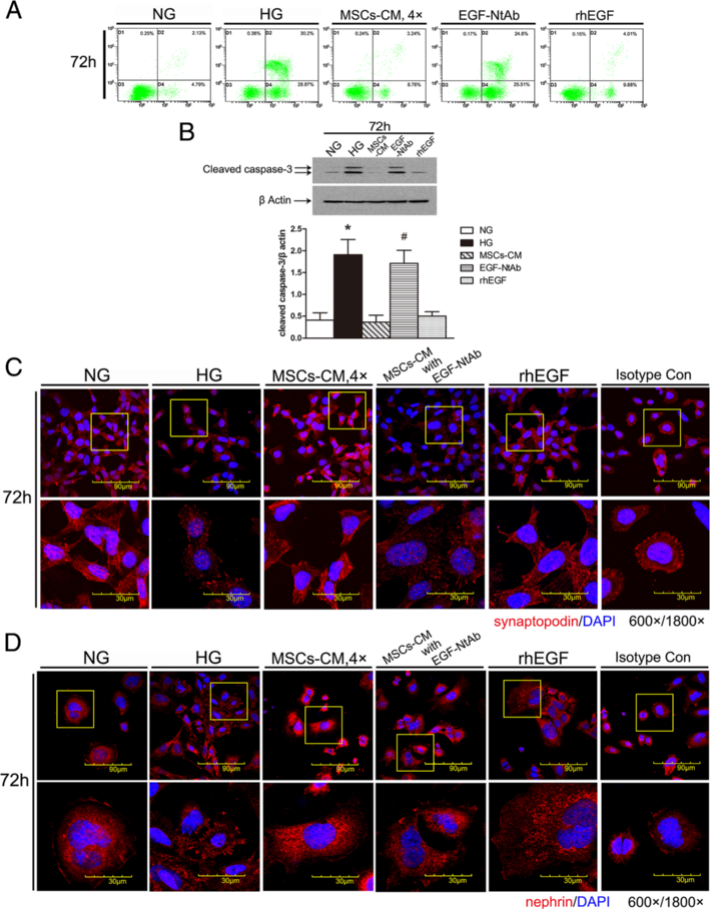
**Epithelial growth factor (EGF) is the main factor in human adipose-derived mesenchymal stem cells (hAd-MSCs)-conditioned medium (CM) to reduce apoptosis and injury of mouse podocyte clone (MPC5) cells induced by high glucose (HG). A)** AnnexinV/PI double staining labeled cells in each group (n = 3 per group) and flow cytometry to determine podocytic apoptosis rate. Recombinant human (rh) EGF group significantly reduced podocytic apoptosis but EGF-neutralizing antibody (NtAb) did not. **B)** Western blot was used to detect the expression of cleaved caspase-3 at three time points (24, 48 and 72 hours). rhEGF reduced the expression of cleaved caspase-3, but EGF-NtAb did not. All of the experiments were repeated three times (n = 3). ^*^P <0.05 HG group *versus* normal glucose (NG) group, MSC-CM group or rhEGF group; ^#^*P* <0.05, EGF-NtAb *versus* NG group, MSC-CM group or rhEGF group. **C)** The expression and the location of the podocytic cytoskeletal protein synaptopodin (red) were measured by confocal microscopy. rhEGF prevented the downregulation and rearrangement of synaptopodin but EGF-NtAb did not. Nuclei were stained with DAPI (blue). Magnification = 600×, 1800×. **D)** The expression of the podocytic membrane protein nephrin (red) was measured by confocal microscopy. rhEGF prevented the downregulation and disorder of nephrin but EGF-NtAb did not. Nuclei were stained with DAPI (blue). Magnification = 600×, 1800 ×. DAPI, 4',6-diamidino-2-phenylindole; PI, propidium iodide.

To verify that EGF was the effector molecule in CM, we cultured MPC5 cells in the presence of HG plus MSC-CM, CM in which EGF was blocked by NtAb, or rhEGF one-component CM. Podocytic apoptosis was detected by annexinV/PI double staining and flow cytometry, cleaved caspase-3 was detected by Western blot and the expression of synaptopodin and nephrin was detected by confocal immunofluorescence. Compared with the HG group, rhEGF significantly prevented podocytic apoptosis induced by HG as shown by the apoptosis rate (*P* <0.05) (Figure 4A) and the expression of cleaved caspase-3 (*P* <0.05) (Figure 4B), and reduced podocytic injury as shown by the expression and the arrangement of synaptopodin and nephrin. However, after blocking EGF in MSC-CM with NtAb, the aforementioned therapeutic effect of MSC-CM was significantly decreased (*P* <0.05) (Figure 4C and D).

## Discussion

Podocytes not only take part in the formation of the filtration barrier but also express many kinds of protein molecules, including synaptopodin and nephrin. The synaptopodin protein of podocytic skelemins is a marker of podocytic differentiation and maturation, which can maintain the normal morphology of podocytes, the normal structure of the glomerular filtration membrane and the charge barrier [24,25]. Some studies have suggested a relationship between the occurrence and development of DN proteinuria and podocytic apoptosis and injury [26,27]. Nephrin is a single-spanning transmembrane immunoglobulin (Ig) superfamily protein and an integral component of the podocyte slit diaphragm, a structure important to the glomerular selectivity filter [28]. Glucose at high levels could reduce the number and density of podocytes, destroy the integrity of the glomerular filtration membrane and change its selective permeability, so as to promote the progression of glomerular sclerosis and accelerate the progress of DN [25,27]. Meanwhile, the loss and injury of interacting proteins of podocytes, such as synaptopodin and nephrin, could activate apoptosis and destroy the integrity of the slit membrane, so as to aggravate proteinuria and accelerate the progression of DN [29]. Consequently, the apoptosis of podocytes and injury of the synaptopodin, nephrin structure play important roles in the occurrence and development of DN. We established an *in vitro* model of podocytic apoptosis and injury induced by HG. We found that HG induced podocytic apoptosis, decreased the expression of synaptopodin, nephrin and rearranged the structure pattern of synaptopodin. These results are consistent with previous reports [7,30].

Recent reports have demonstrated the capacity of MSCs to repair tissue injuries [31]. MSCs transplantation is considered safe and has been widely tested in clinical trials with encouraging results [32]. Our finding [33] and those of other researchers suggest that the effect of MSCs is mediated mainly by cytoprotective, anti-apoptotic, anti-inflammatory factors [34,35], as well as cellular differentiation [36]. In contrast to bone marrow derived-MSCs, Ad-MSCs are more abundant and seem to have several advantages in their differentiation capacity and reparative function [37]. Our previous research found that hAd-MSCs injected via the tail vein reduced kidney injury in a cell localization-independent manner in a type 1 DN rat model. Therefore, we hypothesized that hAd-MSCs may prevent kidney injury through the paracrine action of secreted cytokines. Adding MSCs-CM to the medium of apoptotic podocytes induced by HG, we found the process of apoptosis was inhibited by MSCs-CM, and the decrease and the disorder of podocytic synaptopodin and nephrin were ameliorated by MSCs-CM. It was reported that MSCs can secrete a variety of soluble cytokines to induce the inhibition of apoptosis and repair cell injury, including EGF, GDNF, vascular endothelial growth factor (VEGF), PDGF, IGFBP and IL-x, among others [17,38-40], and these secreted factors can accumulate in CM. It has been reported that MSC-CM has beneficial effects in a variety of tissue repair and cell therapy models, including inhibiting apoptosis and promoting healing and migration [41]. However, we found that WI38 fibroblasts-CM failed to inhibit podocytic apoptosis or injury efficiently. hAd-MSCs are fibroblast-like cells, but Wi38 fibroblasts have no distinct characteristic of stem cells. We speculated that the efficacy of MSCs was mainly due to their ‘stem cell characteristics’, that is, due to the differences between MSCs-CM and WI38-CM.

We then analyzed the differences between MSCs-CM and WI38-CM using an antibody-based cytokine array. The results showed that the levels of IGFBP, GDNF, PIGF and EGF in MSCs-CM were significantly higher than those in WI38-CM but only a dose of EGF equivalent to that in CM could inhibit podocytic apoptosis efficiently. Therefore, we chose EGF as the candidate. EGF can promote the repair and regeneration of damaged epidermis, and in both *in vitro* and *in vivo* experiments EGF can induce anti-apoptosis and repair the damage of epithelial cells, which was also seen in renal tubular epithelial cells, intestinal epithelial cells, and vascular smooth muscle cells [39,40,42]. Whether EGF can inhibit podocytic apoptosis and injury induced by HG has not been reported.

We found that EGF alone could inhibit podocytic apoptosis injury induced by HG but these effects were diminished by blocking EGF with NtAb. We suggest that EGF is necessary for hAD-MSCs to exert their anti-apoptosis and anti-injury therapeutic benefits to podocytic apoptosis and injury induced by HG. One study found that inhibition of EGFR induced the apoptosis of intestinal epithelial cells through an EGF receptor (EGFR)/p38α/mitogen-activated protein kinase (MAPK)/Bax signaling pathway [42]. In addition, Caraglia *et al*. reported that EGF inhibited IFN-α-induced apoptosis in epidermoid tumors by activating the EGF-dependent Ras/extracellular signal-regulated kinase (Erk) signaling pathway [43]. However, Bollee *et al*. reported that epidermal growth factor receptor (EGFR) promotes glomerular injury and renal failure in rapidly progressive crescentic glomerulonephritis [44]. Maybe different mechanisms are involved in these studies. Targeting EGF may have benefit in the diseased kidney. Nevertheless, the mechanism through which EGF secreted into the hAd-MSC-CM to prevent podocytic apoptosis and injury induced by HG needs further study.

## Conclusions

Our findings suggest that hAd-MSCs reduce podocytic apoptosis and injury induced by HG, likely by secreting the cytoprotective factor EGF. To our limited knowledge, there has been no report relevant to the effects of MSCs on podocytic injury induced by HG. Our findings may help to develop a new therapeutic method to ameliorate the progress of DN and shed a new light on the mechanisms of the beneficial effects of MSCs on DN.

## Abbreviations

CM: Conditioned medium; DAPI: 4′,6-diamidino-2-phenylindole; DN: Diabetic nephropathy; EGF: Epithelial growth factor; EGFR: EGF receptor; ELISA: Enzyme-linked immunosorbent assay; FCS: Fetal calf serum; GDNF: Glial cell line-derived neurotrophic factor; hAd-MSCs: Human adipose-derived MSCs; HG: High glucose; HRP: Horseradish peroxidase; IFN: Inhibited interferon; IGFBP: Insulin-like growth factor binding protein; MPC5: Mouse podocyte clone 5; MSCs: Mesenchymal stem cells; NG: Normal glucose; NG+Ma: Mannitol control; NtAb: Neutralizing antibody; OD: Optical density; one-way ANOVA: One-factor analysis of variance; PBS: Phosphate-buffered saline; PI: Propidium iodide; PIGF: Placental growth factor; rhEGF: Recombinant human EGF; RPMI: Roswell Park Memorial Institute; VEGF: Vascular endothelial growth factor; Wi38: Human embryonic lung cells.

## Competing interests

The authors declare that they have no competing interests.

## Authors’ contributions

DGL conceived and designed the experiments, performed the experiments, analyzed the data, and wrote the paper. NW performed the experiments, analyzed the data, and wrote the paper. LZ, HYZ and XYB conceived and designed the experiments and analyzed the data. BF, SYC and WGZ performed the experiments, analyzed the data. XFS performed the experiments and revised the paper. RSL and XMC conceived and revised the paper critically for important intellectual content, and gave final approval of the version to be published. All authors read and approved the final manuscript.

## References

[B1] Shimoi A Hatakeyama H Koizumi H Satoh H Watanabe M Unchanged distribution density of anionic sites on the glomerular wall in rats with streptozotocin-induced diabetic nephropathy Toxicol Pathol 2012 40 789 796 10.1177/0192623312441411 22467625

[B2] Xu ZG Ryu DR Yoo TH Jung DS Kim JJ Kim HJ Choi HY Kim JS Adler SG Natarajan R Han DS Kang SW P-Cadherin is decreased in diabetic glomeruli and in glucose-stimulated podocytes in vivo and in vitro studies Nephrol Dial Transplant 2005 20 524 531 10.1093/ndt/gfh642 15647309

[B3] Isermann B Vinnikov IA Madhusudhan T Herzog S Kashif M Blautzik J Corat MA Zeier M Blessing E Oh J Gerlitz B Berg DT Grinnell BW Chavakis T Esmon CT Weiler H Bierhaus A Nawroth PP Activated protein C protects against diabetic nephropathy by inhibiting endothelial and podocyte apoptosis Nat Med 2007 13 1349 1358 10.1038/nm1667 17982464

[B4] Wang H Madhusudhan T He T Hummel B Schmidt S Vinnikov IA Shahzad K Kashif M Muller-Krebs S Schwenger V Bierhaus A Rudofsky G Nawroth PP Isermann B Low but sustained coagulation activation ameliorates glucose-induced podocyte apoptosis: protective effect of factor V Leiden in diabetic nephropathy Blood 2011 117 5231 5242 10.1182/blood-2010-10-314773 21389321

[B5] Griffin SV Petermann AT Durvasula RV Shankland SJ Podocyte proliferation and differentiation in glomerular disease: role of cell-cycle regulatory proteins Nephrol Dial Transplant 2003 18 vi8 vi13 12953035 10.1093/ndt/gfg1069

[B6] Wang RQ Nan YM Wu WJ Kong LB Han F Zhao SX Kong L Yu J Induction of heme oxygenase-1 protects against nutritional fibrosing steatohepatitis in mice Lipids Health Dis 2011 10 31 10.1186/1476-511X-10-31 21314960 PMC3048569

[B7] Eid AA Gorin Y Fagg BM Maalouf R Barnes JL Block K Abboud HE Mechanisms of podocyte injury in diabetes: role of cytochrome P450 and NADPH oxidases Diabetes 2009 58 1201 1211 10.2337/db08-1536 19208908 PMC2671039

[B8] Maxson S Lopez EA Yoo D Danilkovitch-Miagkova A Leroux MA Concise review: role of mesenchymal stem cells in wound repair Stem Cells Trans Med 2012 1 142 149 10.5966/sctm.2011-0018 PMC3659685 23197761

[B9] Coli A Nocchi F Lamanna R Iorio M Lapi S Urciuoli P Scatena F Giannessi E Stornelli MR Passeri S Isolation and characterization of equine amnion mesenchymal stem cells Cell Biol Int Rep 2011 18 e00011 10.1042/CBR20110004 PMC3475441 23124164

[B10] Chase LG Lakshmipathy U Solchaga LA Rao MS Vemuri MC A novel serum-free medium for the expansion of human mesenchymal stem cells Stem Cell Res Ther 2010 1 8 10.1186/scrt8 20504289 PMC3226302

[B11] Schrijvers BF De Vriese AS Flyvbjerg A From hyperglycemia to diabetic kidney disease: the role of metabolic, hemodynamic, intracellular factors and growth factors/cytokines Endocr Rev 2004 25 971 1010 10.1210/er.2003-0018 15583025

[B12] Bruno S Grange C Deregibus MC Calogero RA Saviozzi S Collino F Morando L Busca A Falda M Bussolati B Tetta C Camussi G Mesenchymal stem cell-derived microvesicles protect against acute tubular injury J Am Soc Nephrol 2009 20 1053 1067 10.1681/ASN.2008070798 19389847 PMC2676194

[B13] Togel F Hu Z Weiss K Isaac J Lange C Westenfelder C Administered mesenchymal stem cells protect against ischemic acute renal failure through differentiation-independent mechanisms Am J Physiol Renal Physiol 2005 289 F31 F42 10.1152/ajprenal.00007.2005 15713913

[B14] Burst V Putsch F Kubacki T Volker LA Bartram MP Muller RU Gillis M Kurschat CE Grundmann F Muller-Ehmsen J Benzing T Teschner S Survival and distribution of injected haematopoietic stem cells in acute kidney injury Nephrol Dial Transplant 2012 28 1131 1139 23197679 10.1093/ndt/gfs513

[B15] Duffield JS Park KM Hsiao LL Kelley VR Scadden DT Ichimura T Bonventre JV Restoration of tubular epithelial cells during repair of the postischemic kidney occurs independently of bone marrow-derived stem cells J Clin Invest 2005 115 1743 1755 10.1172/JCI22593 16007251 PMC1159124

[B16] Yang Z Li K Yan X Dong F Zhao C Amelioration of diabetic retinopathy by engrafted human adipose-derived mesenchymal stem cells in streptozotocin diabetic rats Graefe’s Arch Clin Exp Ophthalmol 2010 248 1415 1422 10.1007/s00417-010-1384-z 20437245

[B17] Salgado AJ Reis RL Sousa NJ Gimble JM Adipose tissue derived stem cells secretome: soluble factors and their roles in regenerative medicine Curr Stem Cell Res Ther 2010 5 103 110 10.2174/157488810791268564 19941460

[B18] Danchuk S Ylostalo JH Hossain F Sorge R Ramsey A Bonvillain RW Lasky JA Bunnell BA Welsh DA Prockop DJ Sullivan DE Human multipotent stromal cells attenuate lipopolysaccharide-induced acute lung injury in mice via secretion of tumor necrosis factor-alpha-induced protein 6 Stem Cell Res Ther 2011 2 27 10.1186/scrt68 21569482 PMC3218818

[B19] Susztak K Raff AC Schiffer M Bottinger EP Glucose-induced reactive oxygen species cause apoptosis of podocytes and podocyte depletion at the onset of diabetic nephropathy Diabetes 2006 55 225 233 10.2337/diabetes.55.01.06.db05-0894 16380497

[B20] Cao Y Sun Z Liao L Meng Y Han Q Zhao RC Human adipose tissue-derived stem cells differentiate into endothelial cells in vitro and improve postnatal neovascularization in vivo Biochem Biophys Res Commun 2005 332 370 379 10.1016/j.bbrc.2005.04.135 15896706

[B21] Chen L Tredget EE Wu PY Wu Y Paracrine factors of mesenchymal stem cells recruit macrophages and endothelial lineage cells and enhance wound healing PloS One 2008 3 e1886 10.1371/journal.pone.0001886 18382669 PMC2270908

[B22] Ben Azouna N Jenhani F Regaya Z Berraeis L Ben Othman T Ducrocq E Domenech J Phenotypical and functional characteristics of mesenchymal stem cells from bone marrow: comparison of culture using different media supplemented with human platelet lysate or fetal bovine serum Stem Cell Res Ther 2012 3 6 10.1186/scrt97 22333342 PMC3340550

[B23] Floege J Kriz W Schulze M Susani M Kerjaschki D Mooney A Couser WG Koch KM Basic fibroblast growth factor augments podocyte injury and induces glomerulosclerosis in rats with experimental membranous nephropathy J Clin Invest 1995 96 2809 2819 10.1172/JCI118351 8675651 PMC185991

[B24] Durvasula RV Shankland SJ Podocyte injury and targeting therapy: an update Curr Opin Nephrol Hypertens 2006 15 1 7 10.1097/01.mnh.0000199012.79670.0b 16340659

[B25] Yanagida-Asanuma E Asanuma K Kim K Donnelly M Young Choi H Hyung Chang J Suetsugu S Tomino Y Takenawa T Faul C Mundel P Synaptopodin protects against proteinuria by disrupting Cdc42:IRSp53:Mena signaling complexes in kidney podocytes Am J Pathol 2007 171 415 427 10.2353/ajpath.2007.070075 17569780 PMC1934530

[B26] Thomas MC Pathogenesis and progression of proteinuria Contrib Nephrol 2011 170 48 56 21659757 10.1159/000324943

[B27] Sakoda M Itoh H Ichihara A Podocytes as a target of prorenin in diabetes Curr Diabetes Rev 2011 7 17 21 10.2174/157339911794273955 21067509

[B28] Wu F Saleem MA Kampik NB Satchwell TJ Williamson RC Blattner SM Ni L Toth T White G Young MT Parker MD Alper SL Wagner CA Toye AM Anion exchanger 1 interacts with nephrin in podocytes J Am Soc Nephrol 2010 21 1456 1467 10.1681/ASN.2009090921 20576809 PMC3013520

[B29] Chen YQ Wang XX Yao XM Zhang DL Yang XF Tian SF Wang NS MicroRNA-195 promotes apoptosis in mouse podocytes via enhanced caspase activity driven by BCL2 insufficiency Am J Nephrol 2011 34 549 559 10.1159/000333809 22123611

[B30] Lee SC Han SH Li JJ Lee SH Jung DS Kwak SJ Kim SH Kim DK Yoo TH Kim JH Chang SH Han DS Kang SW Induction of heme oxygenase-1 protects against podocyte apoptosis under diabetic conditions Kidney Int 2009 76 838 848 10.1038/ki.2009.286 19657327

[B31] Kanazawa H Fujimoto Y Teratani T Iwasaki J Kasahara N Negishi K Tsuruyama T Uemoto S Kobayashi E Bone marrow-derived mesenchymal stem cells ameliorate hepatic ischemia reperfusion injury in a rat model PLoS One 2011 6 e19195 10.1371/journal.pone.0019195 21559442 PMC3084802

[B32] Parekkadan B Milwid JM Mesenchymal stem cells as therapeutics Annu Rev Biomed Eng 2010 12 87 117 10.1146/annurev-bioeng-070909-105309 20415588 PMC3759519

[B33] Wang N Li Q Zhang L Lin H Hu J Li D Shi S Cui S Zhou J Ji J Wan J Cai G Chen X Mesenchymal stem cells attenuate peritoneal injury through secretion of TSG-6 PloS One 2012 7 e43768 10.1371/journal.pone.0043768 22912904 PMC3422344

[B34] Gnecchi M He H Liang OD Melo LG Morello F Mu H Noiseux N Zhang L Pratt RE Ingwall JS Dzau VJ Paracrine action accounts for marked protection of ischemic heart by Akt-modified mesenchymal stem cells Nat Med 2005 11 367 368 10.1038/nm0405-367 15812508

[B35] Mias C Lairez O Trouche E Roncalli J Calise D Seguelas MH Ordener C Piercecchi-Marti MD Auge N Salvayre AN Bourin P Parini A Cussac D Mesenchymal stem cells promote matrix metalloproteinase secretion by cardiac fibroblasts and reduce cardiac ventricular fibrosis after myocardial infarction Stem Cells 2009 27 2734 2743 10.1002/stem.169 19591227

[B36] Yoon BS Moon JH Jun EK Kim J Maeng I Kim JS Lee JH Baik CS Kim A Cho KS Lee HH Whang KY You S Secretory profiles and wound healing effects of human amniotic fluid-derived mesenchymal stem cells Stem Cells Dev 2010 19 887 902 10.1089/scd.2009.0138 19686050

[B37] Hong HS Kim YH Son Y Perspectives on mesenchymal stem cells: tissue repair, immune modulation, and tumor homing Arch Pharm Res 2012 35 201 211 10.1007/s12272-012-0201-0 22370775

[B38] Bailey AM Kapur S Katz AJ Characterization of adipose-derived stem cells: an update Curr Stem Cell Res Ther 2010 5 95 102 10.2174/157488810791268555 19941461

[B39] Lu C Ren W Su XM Chen JQ Wu SH Zhou GP EGF-recruited JunD/c-fos complexes activate CD2AP gene promoter and suppress apoptosis in renal tubular epithelial cells Gene 2009 433 56 64 10.1016/j.gene.2008.11.015 19095050

[B40] Ying WZ Zhang HG Sanders PW EGF receptor activity modulates apoptosis induced by inhibition of the proteasome of vascular smooth muscle cells J Am Soc Nephrol 2007 18 131 142 10.1681/ASN.2006040333 17151333

[B41] Yew TL Hung YT Li HY Chen HW Chen LL Tsai KS Chiou SH Chao KC Huang TF Chen HL Hung SC Enhancement of wound healing by human multipotent stromal cell conditioned medium: the paracrine factors and p38 MAPK activation Cell Transplant 2011 20 693 706 10.3727/096368910X550198 21176394

[B42] Sheng G Guo J Warner BW Epidermal growth factor receptor signaling modulates apoptosis via p38alpha MAPK-dependent activation of Bax in intestinal epithelial cells Am J Physiol Gastrointest Liver Physiol 2007 293 G599 G606 10.1152/ajpgi.00182.2007 17615176

[B43] Caraglia M Tagliaferri P Marra M Giuberti G Budillon A Gennaro ED Pepe S Vitale G Improta S Tassone P Venuta S Bianco AR Abbruzzese A EGF activates an inducible survival response via the RAS- > Erk-1/2 pathway to counteract interferon-alpha-mediated apoptosis in epidermoid cancer cells Cell Death Differ 2003 10 218 229 10.1038/sj.cdd.4401131 12700650

[B44] Bollee G Flamant M Schordan S Fligny C Rumpel E Milon M Schordan E Sabaa N Vandermeersch S Galaup A Rodenas A Casal I Sunnarborg SW Salant DJ Kopp JB Threadgill DW Quaggin SE Dussaule JC Germain S Mesnard L Endlich K Boucheix C Belenfant X Callard P Endlich N Tharaux PL Epidermal growth factor receptor promotes glomerular injury and renal failure in rapidly progressive crescentic glomerulonephritis Nat Med 2011 17 1242 1250 10.1038/nm.2491 21946538 PMC3198052

